# NCKAP1 improves patient outcome and inhibits cell growth by enhancing Rb1/p53 activation in hepatocellular carcinoma

**DOI:** 10.1038/s41419-019-1603-4

**Published:** 2019-05-08

**Authors:** Xiao-ping Zhong, Anna Kan, Yi-hong Ling, Liang-he Lu, Jie Mei, Wei Wei, Shao-hua Li, Rong-ping Guo

**Affiliations:** 1 Department of Burns and Plastic Surgery, The Second Affiliated Hospital of Shantou University Medical College, 515041 Shantou, China; 2 Department of Hepatobiliary Oncology, Sun Yat-sen University Cancer Center, 510060 Guangzhou, China; 3 State Key Laboratory of Oncology in South China, Collaborative Innovation Center for Cancer Medicine, Sun Yat-sen University Cancer Center, 510060 Guangzhou, China

**Keywords:** Liver cancer, Liver cancer

## Abstract

In our previous report, we identified miR-34c-3p as an independent factor contributing to the carcinogenesis of hepatocellular carcinoma (HCC) by targeting NCK Associated Protein 1 (NCKAP1). NCKAP1 has been known to promote the malignancy of cancer cells by disrupting the structural stability of WAS protein family member 1 (WASF1) and is correlated with poor prognosis of patients in several cancer types. Our results, however, show that NCKAP1 is correlated with a favorable outcome in HCC patients. The underlying mechanism of this contradictory phenomenon is unknown. The current study was designed to explore the mechanism of NCKAP1 in HCC. As a result, clinicopathological correlations and results from in vivo and in vitro models indicated that NCKAP1 was a tumor suppressor gene. Cell cycle analysis suggested that NCKAP1 inhibit cells from entering G2/M phase. Western blot analysis showed that WASF1 was barely expressed in HCC cell lines compared to that of breast cancer cell lines, which serve as positive controls. Furthermore, Rb1 and p53 expression was upregulated in cell lines overexpressing NCKAP1. Expression of several cell cycle regulating proteins also varied in the HCC cell lines. In conclusion, although previous studies have identified NCKAP1 as a cell invasion promoter by binding to WASF1, we found that NCKAP1 is a tumor suppress gene that modulates the cell cycle of HCC cell lines by targeting Rb1/p53 regulation.

## Introduction

Hepatocellular carcinoma (HCC) is currently the second leading cause of cancer-related death globally and the number is on the rise^1^. This high mortality rate is contributed to the tumor multiplicity and chromosomal instability of HCC^2^, presenting as intrahepatic and extrahepatic metastases in HCC patients^3^. Thus, methods to decrease the risk of metastasis are needed.

Micro RNAs (miRNAs) posttranscriptional modifications affecting gene expression are well documented^4–6^. Several cancer-related miRNAs and their target genes in tumor cells have been identified^7,8^ and their disposition towards either tumor suppression or oncogenesis clarified^4,6^. In our previous study, we identified a 20-miRNA signature that is significantly associated with the prognosis of HCC patients^9^. Among the 20 miRNAs, we further identified microRNA-34c-3p (miR-34c-3p) as an independent factor contributing to the carcinogenesis of HCC^10^. We also found that miR-34c-3p promotes cell proliferation, colony formation, invasion, and cell cycle progression by targeting NCK Associated Protein 1 (NCKAP1).

NCKAP1 was first discovered in patients with Alzheimer’s disease (AD)^11^. The expression of NCKAP1 is reduced in AD-affected brains^12^. It is likely that the suppression of NCKAP1 induces apoptosis of neuronal cells by interacting with the GTP-binding protein RAC1^13^, a key initiator of actin cytoskeleton reorganization^14^. Furthermore, NCKAP1 has been reported to be an adapter protein binding to RAC1 to activate nucleation through WAS protein family member 1 (WASF1)^15^. In resting cells, WASF1 is maintained in an inactive conformation through its association with a complex of proteins^15^. NCKAP1 is among the protein complex, it is therefore required for WASF1 function and its regulation of invasion. Targeting NCKAP1 is thus reported to lead to the suppression of invasion^16^. WASF1 has been shown to be a promoter of cell invasion in various cancer cell types, including prostate cancer, colorectal cancer, pancreatic cancer, and breast cancer^17–19^. In breast cancer, NCKAP1 is highly expressed and correlates with patient prognosis^20^. NCKAP1 may promote the malignancy of cancer cells by disrupting the structural stability of WASF1^21^. However, the role of NCKAP1 in HCC has not been studied.

In the current study, the aberrant expression of NCKAP1 in HCC tissues was analyzed by quantitative real-time polymerase chain reaction (qPCR) and immunohistochemical (IHC) staining. We demonstrated that the expression NCKAP1 in tumor cells correlated with multiple clinicopathological characteristics and was an independent prognostic factor for HCC patients. In vivo and in vitro assays were performed to determine the function of NCKAP1. Finally, the role of NCKAP1 in malignancy events in HCC cells was also explored.

## Results

### IHC staining of NCKAP1 in HCC tissue specimens and its correlation with clinical pathologic features and survival in HCC patients

**Fig. 1:**
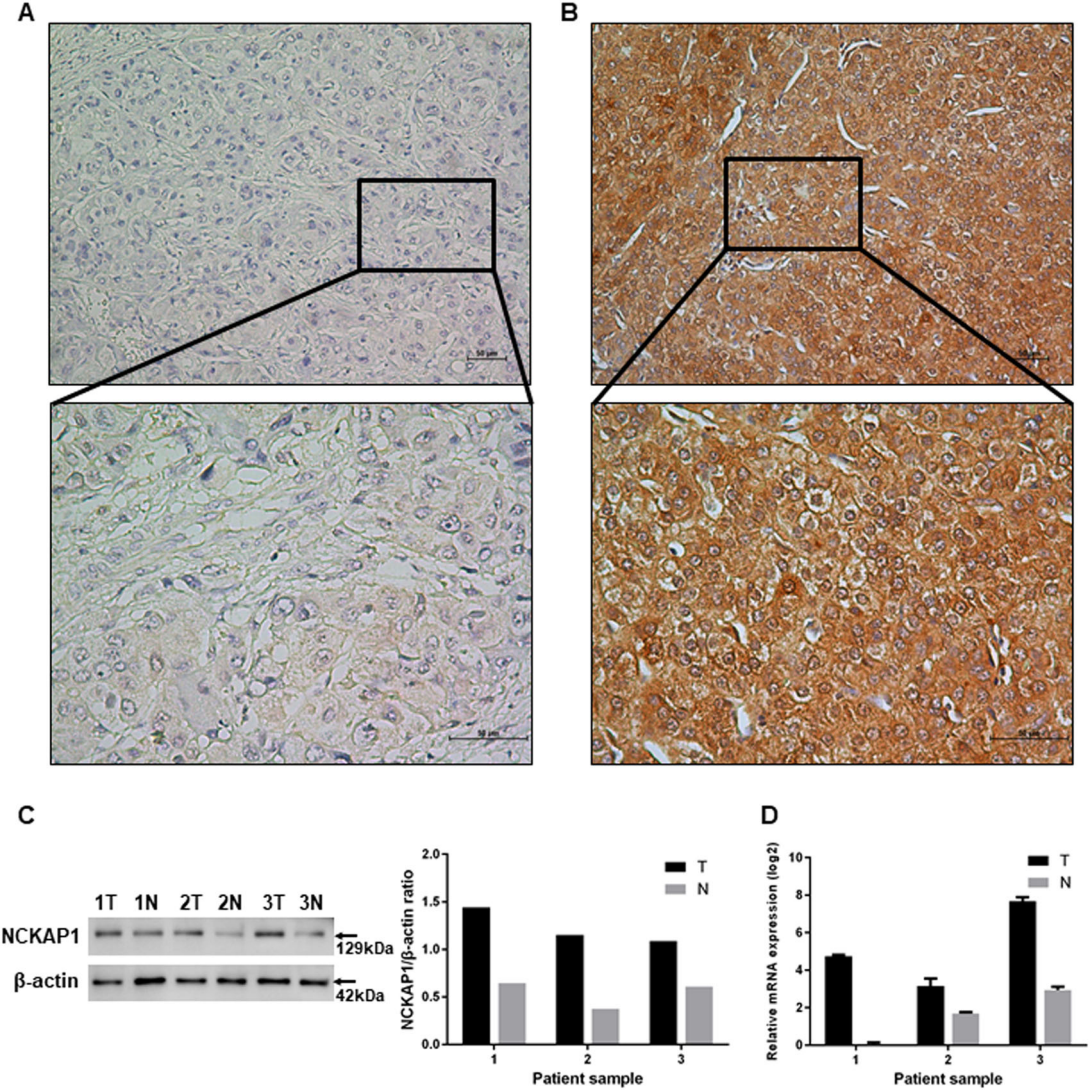
Immunohistochemistry (IHC), western blotting (WB), and quantitative real-time PCR (qPCR) characteristics of NCKAP1 in hepatocellular carcinoma (HCC) specimens. NCKAP1 was primarily expressed in the tumor cell cytoplasm. **a** Representative images of negative NCKAP1 expression in the tumor cytoplasm (×200). The lower panel shows an enlargement of the indicated area (×400). **b** Representative staining of negative NCKAP1 expression in the tumor cell cytoplasm (×200). The lower panel shows an enlargement of the indicated area (×400). **c** WB results show that NCKAP1 exhibited higher expression in tumor tissues compared to adjacent normal tissue in patient samples. **d** qPCR confirms the higher expression of NCKAP1 in tumor tissues

We performed IHC staining of tissue specimens from a set of 201 HCC patients to evaluate the correlation between NCKAP1 expression and HCC prognosis. The results showed that NCKAP1 expression levels in the cytoplasm of tumor cells varied widely among different HCC specimens (Fig. 1a, b). Thus, we focused on the aberrant expression of NCKAP1 in tumor cells. Based on NCKAP1 expression in the tumor cell cytoplasm, patients were divided into two groups, the NCKAP1^−^ group (negative expression in tumor cells; Fig. 1a) and the NCKAP1^+^ group (positive expression in tumor cells; Fig. 1b).

### Elevated expression levels of NCKAP1 in human HCC tissue specimens based on western blot and qPCR analyses

To compare the expression pattern of NCKAP1 in HCC tumor specimens and adjacent nonneoplastic tissues, we examined the levels of NCKAP1 in three pairs of matched HCC tumor-derived and nontumor-derived specimens by qPCR. We found that the expression levels of NCKAP1 were elevated in HCC tumor specimens compared with that of matched adjacent nonneoplastic tissues (Fig. 1c). Moreover, NCKAP1 messenger RNA levels were also significantly higher in HCC tumor tissues compared to that of matched adjacent nonneoplastic tissues (Fig. 1d).

### Effect of NCKAP1 expression on the prognosis of HCC patients in different subgroups

**Fig. 2:**
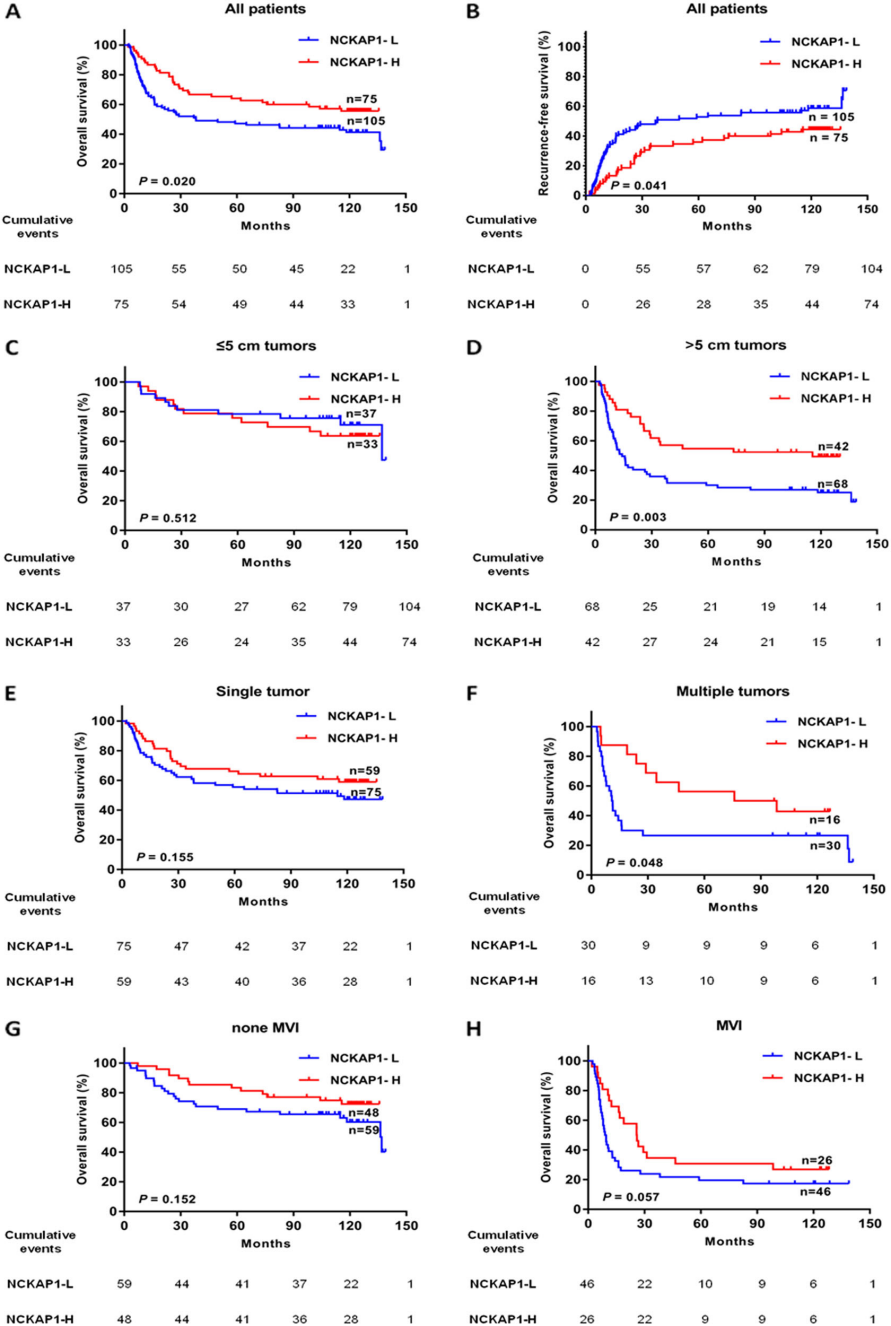
Effect of tumor cell expression of NCKAP1 on the prognoses of all patients and patients stratified into subgroups. **a** Kaplan–Meier survival analysis of overall survival (OS) in all patients. The OS in the NCKAP1-high expression group was significantly increased compared with that in the NCKAP1-low expression group (*P* = 0.02). **b** Kaplan–Meier survival analysis of recurrence free survival (RFS) in all patients. The RFS in the NCKAP1-high expression group was significantly increased compared with that in the NCKAP1-low expression group (*P* = 0.047). **c** Patients with tumor diameter ≤5 cm. **d** Patients with tumor diameter >5 cm. The OS in the NCKAP1-high expression group was significantly increased compared with that in the NCKAP1-low expression group (*P* = 0.003). **e** Patients with a single tumor. **f** Patients with multiple tumors. The OS in the NCKAP1-high expression group was significantly increased compared with that in the NCKAP1-low expression group (*P* = 0.049). **g** Patients without microvascular invasion. **h** Patients with microvascular invasion. The OS in the NCKAP1-high expression group was significantly increased compared with that in the NCKAP1-low expression group (*P* = 0.047)

We analyzed the relationship between NCKAP1 expression levels in the tumor cells and the clinicopathological characteristics (Table 1). To confirm the correlation between NCKAP1 expression levels in tumor cells and HCC prognoses, we compared the time to recurrence and overall survival (OS) between the two groups. Kaplan–Meier survival analysis revealed that patients in the NCKAP1^+^ group had longer mean time to recurrence and better OS than those in the NCKAP1^−^ group (Fig. 2). To determine whether the positive expression of NCKAP1 in tumor cells was an independent prognostic factor for HCC, a multivariate survival analysis was performed. The hazard ratio for OS in the NCKAP1^+^ group was 0.645 (95% confidence interval 0.417–0.997, *P* < 0.05; Table 2). These data indicated that the expression level of NCKAP1 in tumor cells was an independent prognostic factor for HCC. We explored the prognostic value of NCKAP1 in different subgroups of HCC patients. The patients were divided into several subgroups according to tumor diameter, whether single or multiple tumors were present, and microvascular invasion (MVI). The OS of patients with negative NCKAP1 expression was significantly less compared with that of patients with positive NCKAP1 expression in tumors with a diameter >5 cm (*P* < 0.05; Fig. 2d), presenting with multiple tumors (*P* < 0.05; Fig. 2f), and MVI (*P* < 0.05; Fig. 2h).

**Table 1:** Patient baseline and correlation between NCKAP1 expression and clinicopathologic characteristics in hepatocellular carcinoma (HCC)

NCKAP1 expression levels				
Clinicopathological variable	No.	Negative	Positive	*P* value
Age (yr)				0.559
≤50	101	57	44	
>50	79	48	31	
Gender				0.305
Female	19	9	10	
Male	161	96	65	
Hepatitis B surface Ag				0.682
Negative	15	8	7	
Positive	165	97	68	
Serum AFP (ng/mL)				0.325
<400	93	51	42	
≥400	87	54	33	
Tumor size (cm)				0.235
≤5	70	37	33	
>5	110	68	42	
Tumor number				0.272
Solitary	134	75	59	
Multiple	46	30	16	
Microvascular invasion				0.217
No	108	59	49	
Yes	72	46	26	
PVTT				0.916
No	153	89	64	
Yes	27	16	11	
Liver cirrhosis				0.494
No	48	30	18	
Yes	132	75	57	
Differentiation grade				0.467
I + II	112	63	49	
III + IV	68	42	26	
BCLC stage				0.272
0–A	134	75	59	
B–C	46	30	16	
TNM stage				0.405
I	87	48	39	
II–IV	93	57	36	

*AFP* alpha-fetoprotein, *PVTT* portal vein tumor thrombus

**Table 2:** Univariate and multivariate analysis of the association of NCKAP1 with survival and recurrence in patients with hepatocellular carcinoma

Variables	OS			RFS		
	Univariate	Multivariable analysis		Univariate	Multivariable analysis	
	*P* value	HR (95% CI)	*P* value	*P* value	HR (95% CI)	*P* value
Age (yr) (≤50 versus >50)	0.619			0.877		
Gender (female versus male)	0.678			0.981		
Hepatitis B surface Ag (negative versus positive)	0.796			0.713		
Serum AFP (ng/mL) (<400 versus ≥400)	0.026	0.020	1.674 (1.085–2.581)	0.080		
Tumor size (cm) (≤5 versus>5)	0.000	0.000	2.630 (1.620–4.268)	0.000	0.000	2.719 (1.641–4.504)
Tumor number (solitary versus multiple)	0.001	0.161	1.377 (0.880–2.153)	0.000	0.042	1.594 (1.017–2.498)
PVTT (no versus yes)	0.000	0.006	2.170 (1.252–3.762)	0.000	0.060	1.704 (0.978–2.967)
Microvascular invasion (no versus yes)	0.000	0.000	3.067 (1.896–4.960)	0.000	0.000	2.922 (1.793–4.761)
Liver cirrhosis (no versus yes)	0.047	0.021	0.596 (0.385–0.925)	0.110		
Differentiation grade (I + II versus III + IV)	0.036	0.114	1.397 (0.922–2.116)	0.025	0.063	1.489 (0.978–-2.267)
NCKAP1 (negative versus positive)	0.020	0.049	0.645 (0.417–0.997)	0.041	0.234	0.766 (0.493–1.189)

*OS* overall survival, *RFS* recurrence free survival, *AFP* alpha-fetoprotein, *PVTT* portal vein tumor thrombus, *HR* hazard ratio, *CI* confidence interval

### NCKAP1 expression in HCC cell lines and stable transfected cell lines

**Fig. 3:**
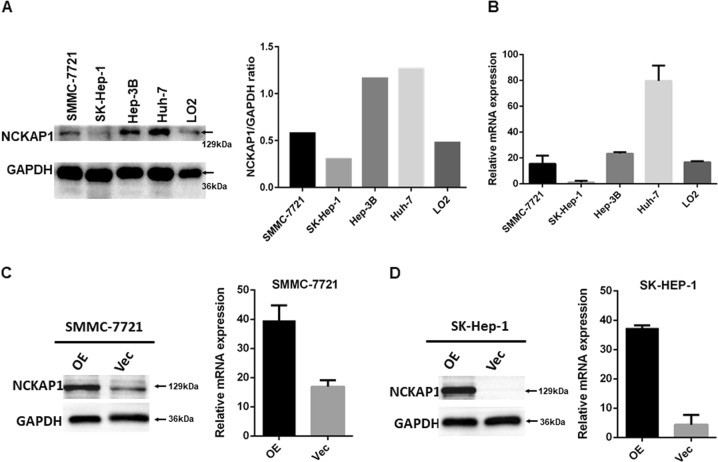
NCKAP1 expression in a normal liver cell line and hepatocellular carcinoma (HCC) cell lines. **a** Western blotting results show that L02, SMMC-7721, and SK-Hep-1 cells exhibited low expression compared to that of Hep-3B and Huh-7 cells. GAPDH was used as a control. **b** Quantitative real-time PCR (qPCR) results confirmed the high expression of NCKAP1 in Hep-3B and Huh-7 cells. **c** Overexpression of NCKAP1 (OE) in a transfected SMMC-7721 cell line verified by western blotting and qPCR compared to that of cells transfected with the control vector (Vec). GAPDH was used as a control. **d** Overexpression of NCKAP1 in a transfected SK-Hep-1 cell line verified by western blotting and qPCR. GAPDH was used as a control

Our results showed that NCKAP1 expression in tumor cells in HCC tissue specimens was negatively associated with malignant clinicopathological features, therefore, we explored the potential biological function of NCKAP1 in HCC tumorigenesis. First, we examined the expression pattern of NCKAP1 in HCC cell lines (Hep3B, SK-Hep-1, Huh7, and SMMC-7721) and normal liver cells (L02). Notably, HCC cell lines SK-Hep-1 and SMMC-7721 displayed significantly lower NCKAP1 messenger RNA and protein levels compared to that of the other HCC cell lines (Fig. 3a, b). To further investigate the role of NCKAP1 in malignancy, SK-Hep-1 and SMMC-7721 cells were stably transfected with an NCKAP1 expression plasmid (pEZ-Lv201-NCKAP1) or a control vector (pEZ-Lv201). The ectopic expression of NCKAP1 messenger RNA and protein in the cells was confirmed by qPCR and western blot analyses, respectively (Fig. 3c, d).

### NCKAP1 displayed an oncogenic function in HCC

**Fig. 4:**
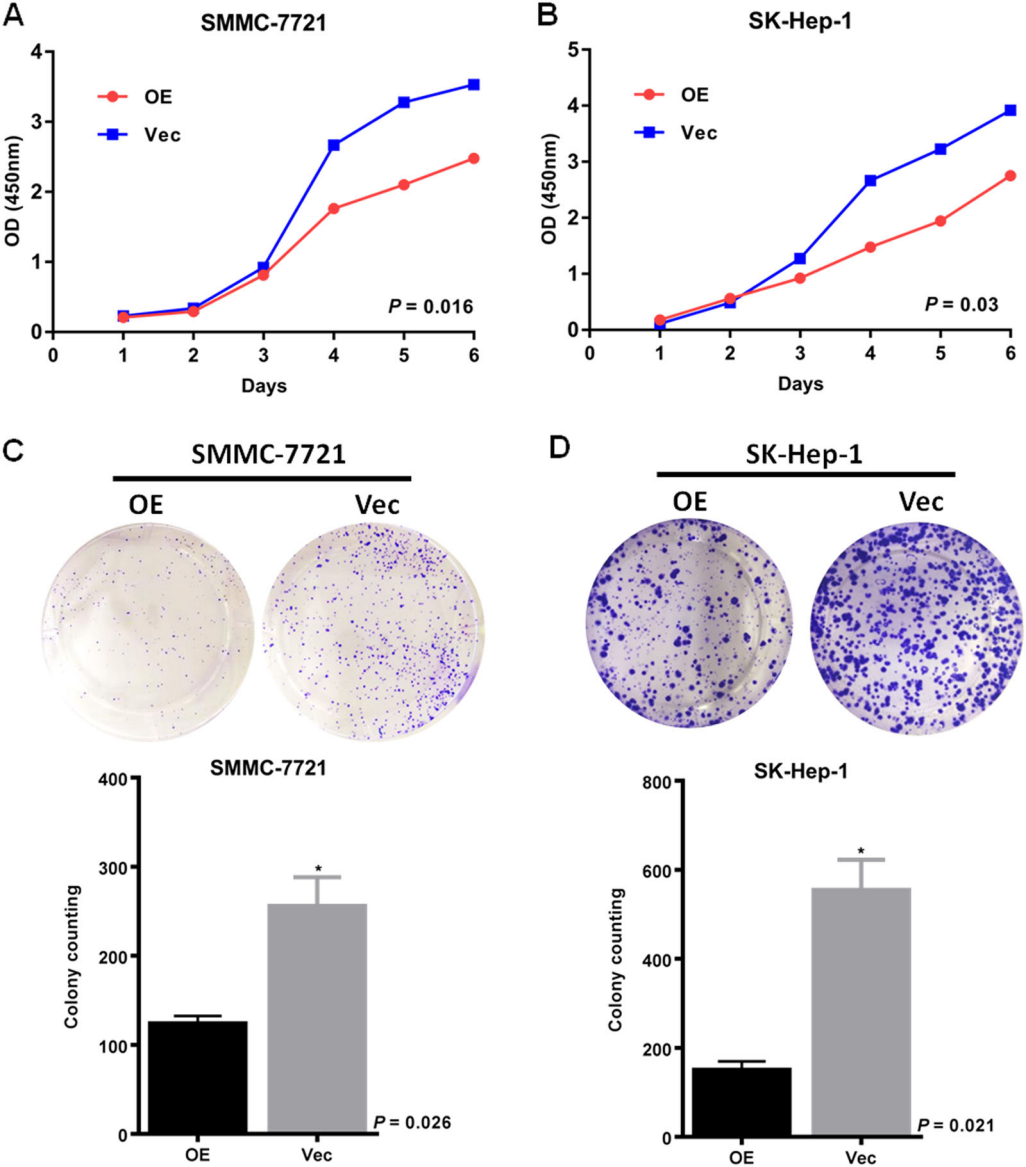
NCKAP1 inhibited cell growth in vitro. **a** CCK8 assay results showing the effect of NCKAP1 overexpression on cell growth in SMMC-7721 cells (SMMC-7721-NCKAP1) compared to that of control cells transfected with control vector (SMMC-7721-Vec). **b** CCK8 assay results showing the effect of NCKAP1 overexpression on cell growth in SK-Hep-1 cells (SK-Hep1-NCKAP1) compared to that of control cells transfected with control vector (SK-Hep-vec). **c** Cell colony formation assay results showing the effect of NCKAP1 overexpression on cell growth in cells (OE) compared to that of control cells transfected with control vector (Vec). **d** Cell colony formation assay results showing the effect of NCKAP1 overexpression on cell growth in SMMC-7721 cells (OE) compared to that of control cells transfected with control vector (Vec)

**Fig. 5:**
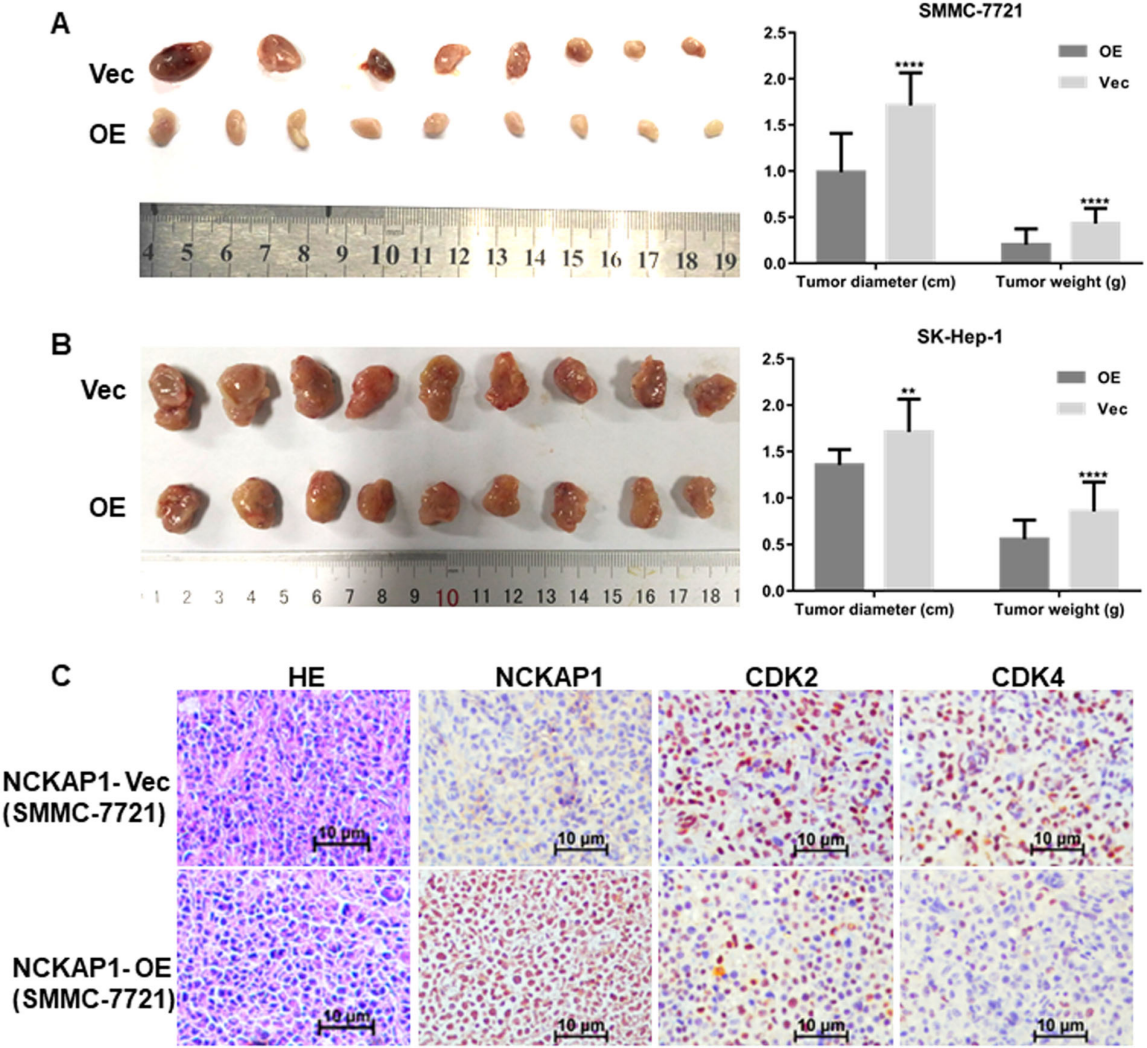
Experimental xenograft mouse models were used to evaluate the effect of NCKAP1 overexpression on tumor growth by subcutaneous injection of NCKAP1-overexpressing cells into BALB/C-nu/nu athymic nude mice. **a** Subcutaneous injection of SMMC-7721-NCKAP1 (NCKAP1-OE) or SMMC-7721-Vector (Control) cells. The tumor diameters and weights are shown in the right panel. **b** Subcutaneous injection of SK-Hep-1-NCKAP1 (OE) or SK-Hep-1-Vector (Control) cells. The tumor diameter and weight are shown in the lower panels. **c** Hematoxylin and eosin (HE) and immunohistochemistry (IHC) staining showed that the expression of NCKAP1, CDK2, and CDK4 differed

Functional assays were used to characterize the tumorigenicity of NCKAP1. The results demonstrated that overexpression of NCKAP1 in HCC cell lines significantly inhibited the rate of cell growth (Fig. 4a, b) and frequency of foci formation (Fig. 4c, d) compared to those in the control cells. To determine function of NCKAP1 in vivo, transfected cells overexpressing NCKAP1 or vector-control cells were subcutaneously injected into nude mice. At 4 weeks post grafting, the mice were sacrificed and the xenograft tumors were harvested and measured. The results showed that the xenograft tumors of the NCKAP1 overexpression group were significantly smaller and less frequent (*P* < 0.05) compared to those of the control group (Fig. 5a, b). Morphological changes were assessed by HE staining. Compared to the control group, SMMC-7721 cells in the NCKAP1 overexpression group showed chromatin condensation and nucleus fragmentation, and apoptotic degree increased, as shown in Fig. 5c. The expression of NCKAP1, CDK2, and CDK4 also differed in IHC analysis performed on sectioned subcutaneous tumors from BALB/C-nu/nu athymic nude mice in Fig. 5c.

### NCKAP1 modulated cell apoptosis and cell cycle in HCC

**Fig. 6:**
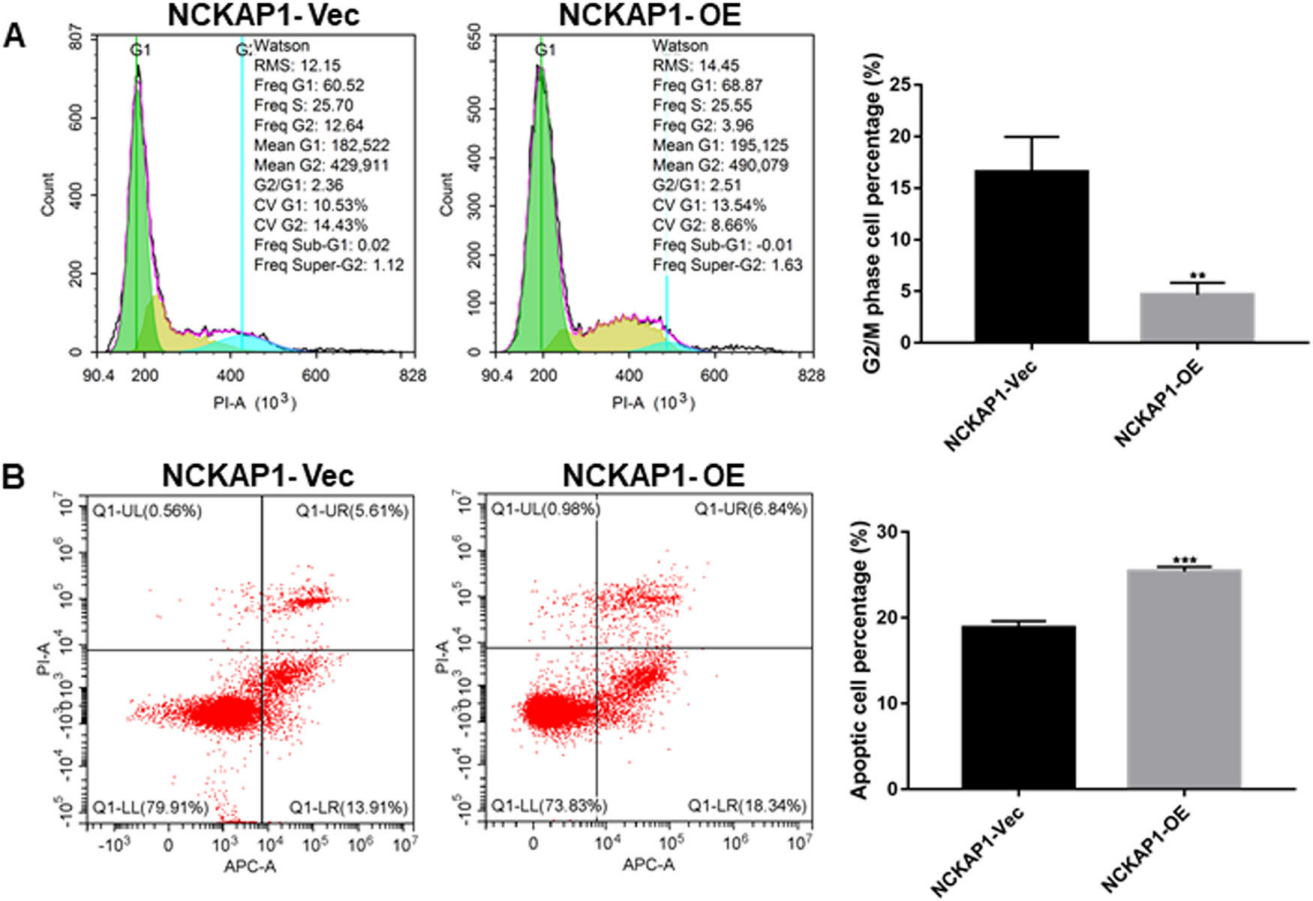
NCKAP1 promote cell death in vitro. **a** Cell cycle assessment of in OE cells showing a decrease of cells in G2 phase compared to that of control cells transfected with control vector (Vec). **b** Annexin V staining showing the accumulation of early and late apoptotic cells in the OE group compared to that of control cells transfected with control vector (Vec)

The cell apoptosis and cell cycle status of HCC cell lines were analyzed by flow cytometry. As shown in Fig. 6a, the proportion of NCKAP1-OE cells in G2/M phase was significantly lower compared with that in the control group, suggesting that NCKAP1 inhibit cells from entering G2/M phase (Fig. 6a). In addition, flow cytometry showed that the percentage of apoptotic cells in the NCKAP1 overexpression group was significantly higher compared to that in the control group (Fig. 6b).

### WASF1 expression in HCC cells

**Fig. 7:**
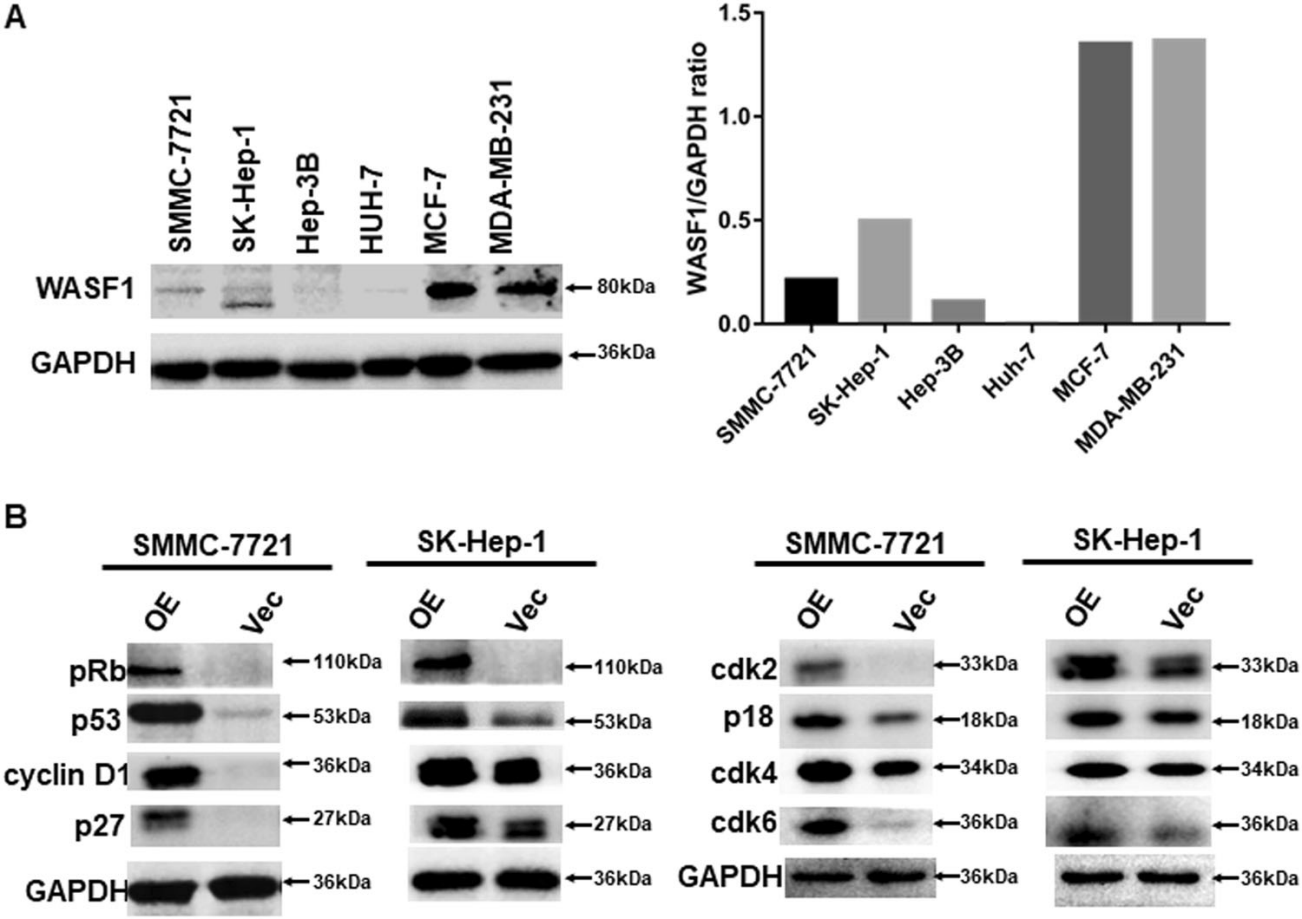
WASF1, Rb1, and p53 expression in hepatocellular carcinoma (HCC) and breast cancer cell lines. **a** Western blotting results show that HCC cell lines (7721, SK, 3B, and HUH7) exhibited fairly low expression of WASF1 compared to that of breast cancer cell lines (MC-7 and MDA-MB-231). GAPDH was used as a control. **b** NCKAP1-overexpressing (OE) HCC cell lines (SMMC-7721 and SK-Hep-1) showed upregulated expression of Rb1, p53, cyclin D1, p27, cdk2, p18, cdk4, and cdk6 compared to that of cells transfected with the vector control (Vec), as detected by western blotting. GAPDH was used as a control

It has been extensively reported that the WASF1 complex is steadily expressed in breast cancer cell lines and is essential for the invasion of breast cancer^19,20,22,23^. Therefore, we obtained two breast cancer cell lines, MCF-7 and MDA-MB-231, which were used as positive controls for examining WASF1 expression in HCC cell lines. The result demonstrated that WASF1 was only minimally expressed in HCC cell lines compared to that of breast cancer cell lines (Fig. 7a).

### NCKAP1 expression induced the Rb1 pathway in HCC cells

It is well known that inactivation of the Rb1 pathway leads to cancer^24–27^. In addition, the cooperation of Rb1 and p53 may suppress cancer malignancy by regulating the cell cycle^26^. In the current study, we observed that oncogenic functions were inhibited in NCKAP1-transfected cells (Figs. 4–6). We therefore examined the expression of Rb1, p53, and several cell cycle regulation proteins in NCKAP1-transfected HCC cell lines. Results of western blotting showed that overexpression of NCKAP1 in SK-Hep-1 and SMMC-7721 cells markedly enhanced the levels of Rb1 and p53 (Fig. 7b). Meanwhile, expression of the cell cycle regulating proteins cyclin D1, p27, CDK2, p18, CDK4, and CDK6 varied in the HCC cell lines (Fig. 7b).

## Discussion

In our previous study, we found that miR-34c-3p is an independent factor contributing to the carcinogenesis of HCC. We also found an inverse correlation between IHC staining scores for NCKAP1 and miR-34c-3p expression levels in 33 HCC specimens (*P* < 0.001)^10^. In the current study, we further defined the frequent aberrant expression of NCKAP1 in HCC tissue specimens using qPCR and IHC. The expression pattern of NCKAP1 was negatively associated with malignant clinicopathological characteristics. More significantly, we found that the NCKAP1 expression pattern in HCC tissue specimens was associated with better prognoses following curative surgery. Furthermore, multivariate analyses revealed that NCKAP1 expression in tumor cells was an independent risk factor affecting recurrence and survival following curative surgery.

Previous studies showed NCKAP1 to be a cancer promoting factor in cancer patients, leading to poor prognosis. In the current study, we set out to determine the underlying mechanism of NCKAP1 in promoting HCC. Our IHC results were consistent with previous studies showing significantly higher expression levels of NCKAP1 compared to that of normal paratumorous tissue^10^. However, statistical analyses revealed that higher expression of NCKAP1 correlated with better outcome for HCC patients. This finding conflicted previous results. Furthermore, cell functional assays showed similar conflicting results in which cell lines overexpressing NCKAP1 demonstrated suppressed cell migration and invasion, while knockdown of NCKAP1 inhibited the rate of cell growth and frequency of foci formation. Our results were contradictory to those from previous studies, suggesting that NCKAP1 may play a different role in HCC compared to that in other cell types^20,21^.

To verify our suspicion, we performed western blotting and qPCR to examine the expression of WASF1 in HCC cell lines and used breast cancer cell lines as positive control groups. We found that compared to breast cancer cell lines, the expression of WASF1 was significantly lower in HCC cells, suggesting that WASF1 may not be involved in HCC malignancy. This finding corroborated our hypothesis. Therefore, NCKAP1 may function through a different pathway in HCC than that in other cancers. We then evaluated the results from the cell function assays. Our results suggested that NCKAP1 may have inhibited HCC oncogenic behavior, which may be correlated with the stimulation of the Rb1 pathway.

**Fig. 8:**
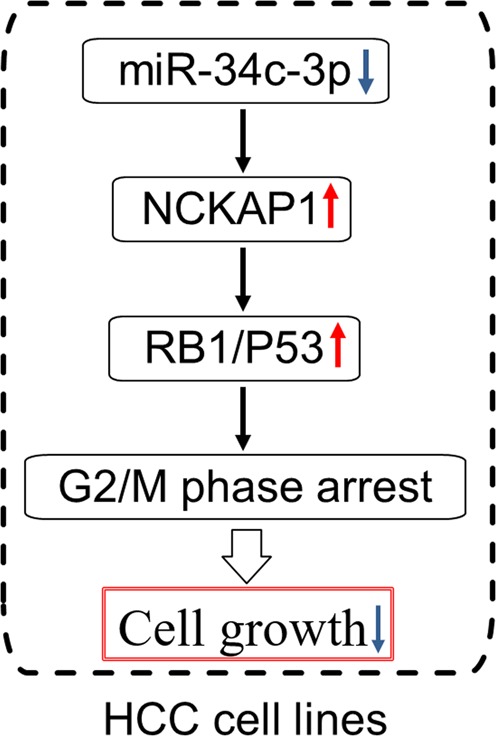
A diagrammatic schematic sketch of miR-34c-3p/NCKAP1/RB1/P53 controlling cell cycle and proliferation in this study. Downregulation of miR-34c-3p leads to elevated expression of NCKAP1, which arrests the cell cycle in G2/M phase through RB1/P53 pathway and inhibits cell growth in HCC cell lines

The Rb1 pathway is known to be a negative regulator of the cell cycle^28^. It has been reported that the loss of Rb1-mediated cell cycle control is frequently observed in cancer^29^. Moreover, the loss of Rb1 has profound effects on many other cellular processes relevant to cancer, including differentiation^30^. It has also been reported that Rb1 and p53 cooperate to repress epigenetic reprogramming factors in prostate cancer^26^. Ku et al. demonstrated that in prostate adenocarcinoma, Rb1 loss facilitates lineage plasticity and metastasis and that the additional loss of p53 causes resistance to therapy^31^. However, there have not been similar reports on NCKAP1/Rb1/p53 pathway in HCC. Our results from flow cytometry demonstrated that a high percentage of NCKAP1 overexpression cells were arrested in G2/M phase, suggesting that NCKAP1 was a significant regulator of the cell cycle. We therefore examined the expressions of Rb1, p53, and several cell cycle proteins in HCC cell lines overexpressing NCKAP1. The results showed that following NCKAP1 overexpression, Rb1 and p53 were correspondingly dysregulated. The expression of cell cycle regulating proteins cyclin D1, p27, CDK2, p18, CDK4, and CDK6 was also altered. This suggested that NCKAP1 may regulate HCC oncogenic behavior in response to the functions of Rb1 and p53 and thereby modulate the cell cycle of HCC (Fig. 8).

## Conclusions

In conclusion, previous studies have identified NCKAP1 as a key component binding to a complex of proteins known as the WASF regulatory complex, which functions as a promoter of cell invasion in several cell lines^17–19^. However, our current study was the first to explore the role of NCKAP1 in HCC. We found that WASF1 was hardly expressed in HCC and therefore its function may be limited. Hence, NCKAP1 may function in an entirely different matter compared to that in other types of cancer. This suspicion was verified by several in vivo and in vitro experiments. Our study suggested that NCKAP1 was a tumor suppressor gene that modulated the cell cycle of HCC by targeting p53. However, this study merely highlighted that NCKAP1 played a role in cell cycle modulation, and did not ascribe the upstream or downstream functions of this particular gene. Therefore, while our findings provide important new insight, the mechanism of NCKAP1 requires further investigation.

## Materials and methods

### Patients and tissue specimens

Formalin-fixed, paraffin-embedded tissues were obtained from 201 patients, who underwent curative resection for HCC at Sun Yat-sen Cancer Center between 2006 and 2008. The inclusion criteria used for patient enrollment was an absence of anticancer therapies or distant metastasis prior to the surgery; a lack of concurrent autoimmune disease, human immunodeficiency virus, or syphilis; and the availability of follow-up data. Patients with Child–Pugh class B or C were excluded from the study. The histologic grade of tumor differentiation was assigned according to the Edmondson–Steiner grading system. The BCLC staging system and the seventh edition of the International Union Against Cancer/American Joint Committee on Cancer TNM staging system were used for staging. The clinicopathological characteristics of the patients are summarized in Table 1. An additional three pairs of freshly resected HCC specimens and adjacent nonneoplastic liver tissues were collected from patients who had undergone hepatectomies for curative treatment of HCC at the Sun Yat-sen Cancer Center in 2017. None of these three patients received any neoadjuvant therapies before surgery, including radiotherapy or chemotherapy. This study was approved by the institutional review boards of the Sun Yat-sen Cancer Center. Written informed consent was obtained from all patients, including for the use of their liver specimens for research.

### Follow-up

During follow-up, serum alpha-fetoprotein (AFP) levels were monitored and three-phase dynamic computed tomography scan or magnetic resonance imaging was performed every one to three months after surgery. The OS time was defined as the interval between surgery and death or between surgery and the last follow-up for surviving patients. The time to recurrence was defined as the interval between surgery and recurrence or between surgery and the last follow-up for patients without recurrence. The median follow-up was 55 mo (range 2–84 mo). Of the 180 patients examined during the follow-up period, 83 patients (46.1 %) died and 89 patients (49.4 %) were diagnosed with tumor recurrence.

### Statistical analyses

For continuous variables, the data are expressed as the mean ± standard error of the mean. The significance of differences between values was determined using the Student’s *t*-test. The chi-squared test was applied to examine the correlation between NCKAP1 expression and clinical pathological parameters. Survival curves for patients were calculated using the Kaplan–Meier method and analyzed using the log-rank test. Prognostic factors were examined by univariate and multivariate analyses using the Cox proportional hazards model. All differences were deemed significant at *P* < 0.05. All statistical analyses were performed using SPSS software version 19.0 (SPSS, Chicago, IL).

### Cell lines and culture conditions

The human hepatocellular carcinoma (HCC) cell lines SK-Hep-1 and SMMC-7721, the normal hepatic cell line L02, and the human embryonic kidney 293T (HEK 293T) cell line were obtained from the Liver Cancer Institute of Fudan University (Shanghai, China). The breast cancer cell lines MCF-7 and MDA-MB-231 were obtained from the American Type Culture Collection (ATCC, Manassas, VA, USA). All cell lines were cultured in Dulbecco’s modified Eagle’s medium (DMEM; Gibco, Carlsbad, CA, USA) supplemented with 10% fetal bovine serum (FBS; Gibco). The cells were incubated at 37 °C in a humidified incubator supplied with 5% carbon dioxide.

### Plasmid constructs and transfection of cell lines

Human NCKAP1 complementary DNA (cDNA) was cloned into the pEZ-Lv201 vector (GeneCopoeia, Inc., Rockville, MD, USA) and then transfected into the 293T packaging cell line using a Lenti-Pac™ HIV Expression Packaging Kit (GeneCopoeia, Inc.) according to the manufacturer’s instructions. Virus-containing supernatants from the transfected 293T cells were collected and filtered using 0.45-μm filters. The filtered supernatant was added to 70%-confluent cells in the presence of 8 μg/mL polybrene (Sigma, St. Louis, MO, USA). After 48 h, the cells were incubated with fresh complete medium containing the appropriate concentration of puromycin for stable transduced cells. Empty pEZ-Lv201 was transfected as a vector control in the same manner.

### Immunohistochemistry

Paraffin-embedded samples were cut as 5-μm sections and processed for immunohistochemistry. Tissue sections were prepared for antigen retrieval using microwave treatment in citrate buffer (pH 6.0) and then incubated with anti-NCKAP1 antibody (Proteintech, Rosemont, IL, USA), anti-CDK2 antibody (Cell Signaling Technology, Danvers, MA, USA), or anti-CDK4 antibody (Cell Signaling Technology). Immunostaining was performed using the Envision System with diaminobenzidine as substrate (Dako Cytomation, Glostrup, Denmark). Images were viewed and assessed using an Eclipse 80i microscope (Nikon, Tokyo, Japan). Hematoxylin and eosin (H&E) staining was performed using a Hematoxylin 7211 and Eosin-Y Alcoholic kit (Thermo Fisher Scientific, Inc, Shanghai, China).

### Western blot analysis

Tissues and cells were lysed on ice in 50 mM Tris (pH 7.5), 150 mM NaCl, and 0.5% NP-40. Protein lysates were separated using 10% sodium dodecyl sulphate-polyacrylamide gel electrophoresis and then transferred to a polyvinylidene fluoride membranes. After the membranes were blocked with 5% bovine serum albumin, they were incubated with the various primary antibodies at 4 °C overnight. The membranes were then incubated with horseradish peroxidase-conjugated secondary antibodies at room temperature for 45 min. Protein signals were detected using enhanced chemiluminescence (Pierce, Rockford, IL, USA). Bands were quantified using ImageJ (Ver. 1.52a; NIH, Bethesda, MD), normalized to GAPDH, and the ratios were determined.

### Antibodies and reagents

Rabbit anti-NCKAP1 was purchased from Proteintech. Rabbit anti-WASF1, anti-pRb, anti-P53, anti-cyclin D1, anti-p27, anti-CDK2, and anti-CDK4 antibodies and mouse anti-p18 and anti-CDK6 antibodies were purchased from Cell Signaling Technology.

### RNA isolation and quantitative real-time PCR

Total RNA was isolated from the tissue specimens and cell lines using TRIzol Reagent (Invitrogen Life Technologies, Shanghai, China). The isolated RNA (2 μg) was reverse-transcribed to cDNA using a SuperScript^®^ III First-Strand Synthesis System (Invitrogen Life Technologies) according to the manufacturer’s instructions. For the qPCR assay, the cDNA was subjected to PCR amplification using SYBR Green (Toyobo, Kita-ku, Osaka, Japan) and a Roche LightCycler 480 System. GAPDH was used as an internal control. The primers used included the NCKAP1 forward primer (5′-TCCTAAATACTGACGCTACAGCA-3′), NCKAP1 reverse primer (5′-GCCTCCTTGCATTCTCTTATGTC-3′), GAPDH forward primer (5′-GGTATGACAACGAATTTGGC-3′), and GAPDH reverse primer (5′-GAGCACAGGGTACTTTATTG-3′).

### In vitro cell growth assays

The proliferative activity of cells was determined using Cell Counting Kit-8 (CCK8) assays (Promega, WI, USA) with the number of viable cells being evaluated after 2 h in medium containing CCK8. The conversion of the tetrazolium salt WST-8 to formazan was measured at 450 nm using a plate reader. For colony formation assays, 1 × 10^3^ cells were seeded in each well of six-well plates and cultured with DMEM (Gibco) supplemented with 10% FBS (Gibco) for 7 days. The colonies were washed twice with phosphate-buffered saline, fixed in methanol for 15 min, and stained with crystal violet for 15 min at room temperature. After the excess stain was washed out, the number of colonies was counted. Colony formation efficiency was calculated as the ratio of the number of colonies formed to the total number of cells plated.

### Cell migration and invasion assays

Cell migration and invasion assays were performed in Transwell^®^ chambers (8-μm pore size; Corning, NY, USA). For the migration assays, 1 × 10^4^ cells were seeded into the upper chamber of each insert. For the invasion assays, 1 × 10^5^ cells with 1:6 diluted Matrigel (BD Biosciences, San Jose, CA, USA) were seeded into the upper chamber of each insert. The upper chambers were supplemented with serum-free medium and as a chemo-attractant medium with 5% FBS was added to the lower chambers. The tumor cells were cultured at 37 °C for 24 h. Five random fields were counted in triplicate assays.

### Annexin V assay

Annexin V binding assays were performed using an Annexin V Detection Kit (4A Biotech Co., Beijing, China) according to the manufacturer’s instructions. Briefly, 5 × 10^5^ cells were seeded into six-well tissue culture plates. The cells were cultured in DMEM (Gibco) supplemented with 10% FBS (Gibco). The cells were harvested when confluency reached 70–80%, washed twice with wash buffer, and incubated with 0.25 µg/mL annexin V reagent in 1× binding buffer for 15 min. The cells were washed twice to remove excess annexin V and were resuspended in binding buffer containing 0.5 µg/mL propidium iodide. A flow cytometer was used to acquire 20,000 events. Early and late phase apoptotic cells were segregated and presented in a quadric plot graph.

### Cell cycle analysis

Briefly, 5 × 10^5^ cells were added to each well of a six-well tissue culture plate. When confluency reached 70–80%, the cells were fixed in cold 70% ethanol and placed overnight at 4 °C. The cells were resuspended and incubated with RNaseA solution at 37 °C for 30 min, and then PI solution was added to the cell suspension incubated for another 30 min at 4 °C. A flow cytometer was used to determine the proportion of cells in G1 phase, S phase, and G2 phase.

### In vivo tumorigenesis assays

All the animal experiments were performed in accordance with the guidelines of the Laboratory Animal Ethics Committee of Sun Yat-Sen University. For the in vivo tumorigenesis model, 40 four-week-old BALB/C-nu/nu athymic nude mice were divided into four groups. The BALB/C-nu/nu athymic nude mice were then injected subcutaneously with 5 × 10^6^ cells transfected with SK-NCKAP1, SK-vector, 7721-NCKAP1, or 7721-vector. At 4 weeks post grafting, the subcutaneous tumors were resected, fixed in phosphate-buffered neutral formalin, sectioned serially, and stained with hematoxylin-eosin. IHC analysis was then performed on separate sections.

## Supplementary information

SUPPLEMENTAL MATERIAL 1

## Data Availability

The datasets supporting the conclusions of this article are available in the Research Data Deposit repository, http://www.researchdata.org.cn/. The RDDN of the dataset for this study is RDDB2018000352.
