# Supplementary figures and images for: NCKAP1 improves patient outcome and inhibits cell growth by enhancing Rb1/p53 activation in hepatocellular carcinoma
Source: Cell Death Dis. 2019 May 8;10(5):369. doi: 10.1038/s41419-019-1603-4 (PMC6506474; doi:10.1038/s41419-019-1603-4)

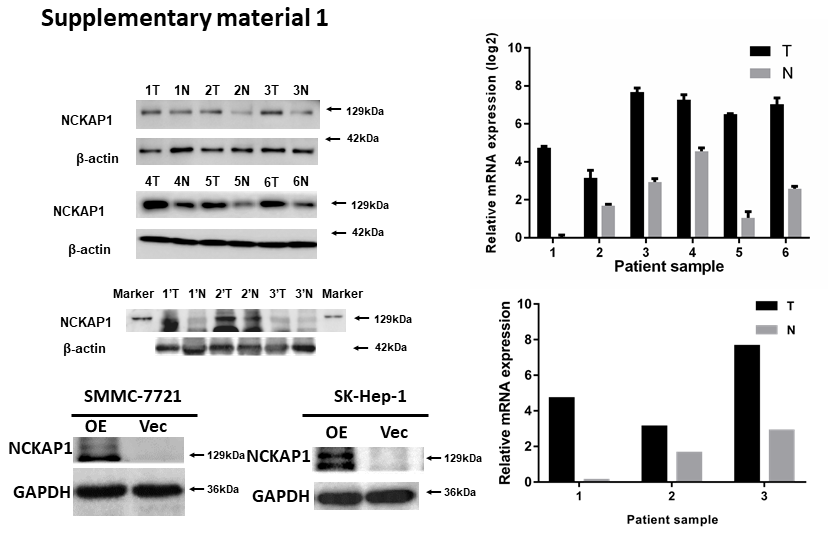

Supplement: Supplementary file 1 — SUPPLEMENTAL MATERIAL 1 [file 41419_2019_1603_MOESM1_ESM.tif]
